# The smartphone camera as a potential method for transcutaneous bilirubin measurement

**DOI:** 10.1371/journal.pone.0197938

**Published:** 2018-06-01

**Authors:** Sarah B. Munkholm, Tobias Krøgholt, Finn Ebbesen, Pal B. Szecsi, Søren R. Kristensen

**Affiliations:** 1 Department of Acute Medicine, Randers Regional Hospital, Randers, Denmark; 2 Aalborg University, Aalborg, Denmark; 3 Department of Paediatrics, Aalborg University Hospital, Aalborg, Denmark; 4 Department of Clinical Biochemistry, Copenhagen University Hospital Holbæk, Holbæk, Denmark; 5 Department of Clinical Biochemistry, Aalborg University Hospital, Aalborg, Denmark; Laval University, CANADA

## Abstract

**Background:**

Hyperbilirubinemia is a common problem in neonates that can progress into kernicterus. Suspected neonatal hyperbilirubinemia is a common reason for contact with the healthcare system. The severity and management of jaundice are determined based on estimated bilirubin levels. However, no easy and accessible tool for self-assessing neonatal jaundice is currently available. Smartphones could potentially be transformed into a medical device that could be used by both patients and practitioners.

**Objective:**

To investigate whether a digital image produced by a camera embedded on a smartphone can be a used as a screening tool for neonatal hyperbilirubinemia.

**Study design:**

A total of 64 randomly selected newborns were enrolled. The inclusion criteria were healthy Caucasians, gestational age >35 weeks, age >24 hours and ≤14 days old, and parental informed consent. The exclusion criteria were facial skin lesions and light treatment. Images of the glabella were obtained with an iPhone 6 via i) directly applied pressure, ii) a dermatoscope, or iii) a dermatoscope equipped with a Wratten No. 11 filter. The red, green and blue colour intensities of each image were compared to bilirubin levels.

**Results:**

Only the dermatoscope-acquired intensities of the green and blue channels were significantly correlated (*p* < 0.001) with bilirubin measurements (Pearson’s *r*: 0.59 and 0.48, respectively). For the green and blue channels, discrimination limits of 212 and 190, respectively, revealed a sensitivity and specificity of 100% and 62.5%, respectively, for green and 90.9% and 60%, respectively, for blue for a plasma bilirubin above 205 μmol/L.

**Conclusions:**

The results of this study indicate that a smartphone equipped with a consistent light source in the form of a dermatoscope may be a simple screening tool for neonatal hyperbilirubinemia. However, the method requires some improvement before clinical application.

## Introduction

About half of term newborns develop hyperbilirubinemia, but bilirubin reaches levels that cause acute or chronic encephalopathy (kernicterus) only in rare cases [1]. This condition is treatable with phototherapy or, very rarely, exchange transfusion [2–4]. Bilirubin levels in term neonates peak within three to five days after birth, and most infants have already been discharged by this time. Thus, the parents are frequently alarmed by a suspicion of jaundice, which is therefore a common cause of admission and readmission in the healthcare system. Alternatively, hyperbilirubinemia may not be recognised, resulting in potential complications. In less developed countries with more difficult access to medical facilities, this is even a bigger issue.

Various methods are available to assess bilirubin levels in neonates. These include visual assessment, non-invasive transcutaneous bilirubinometers (TcB) and determination of total plasma bilirubin (TsB) levels. The simplest method, the subjective visual assessment of skin colour, is often the first method used to evaluate an infant’s status. However, this method is not reliable even when performed by experienced healthcare professionals [5]. TsB levels are the gold standard method for assessing bilirubin levels in neonates [4,6–8], and it therefore remains one of the most frequently performed tests in neonates, resulting in costly and invasive blood sampling and an increased risk of infection [9], pain [10] and parental stress [11]. A TcB can be used as a screening tool to reduce the frequency of blood sampling [12,13]. None of these tools are available as point-of-care tests because they are too expensive to use for home monitoring [6].

Several approaches have been explored in the past years to substitute or support the current instruments [14,15]. Only one has shown significant results [16]. Smartphones have cameras, and the colours in a picture can be analysed to determine the intensity of the three colours red, green and blue. Since high bilirubin levels result in yellowish colouring of the skin, an analysis of the skin colour of a baby could potentially be used to determine the level of bilirubin.

In this study, we aimed to investigate whether a correlation analysis of a digital image produced by a camera embedded on a smartphone can be used as a screening tool for neonatal hyperbilirubinemia.

## Materials and methods

### Subjects

A total of 64 newborns were randomly selected and enrolled in the study when sampling for phenylketonuria screening was performed at Aalborg University Hospital in Denmark between the 15th of July 2016 and the 15th of June 2017. The inclusion criteria were healthy Caucasians, gestational age at birth ≥35 weeks, and age >24 hours and ≤14 days old. The exclusion criteria were facial skin lesions on the forehead and phototherapy for hyperbilirubinemia. All demographic and clinical data were obtained from hospital records.

**Fig 1:**
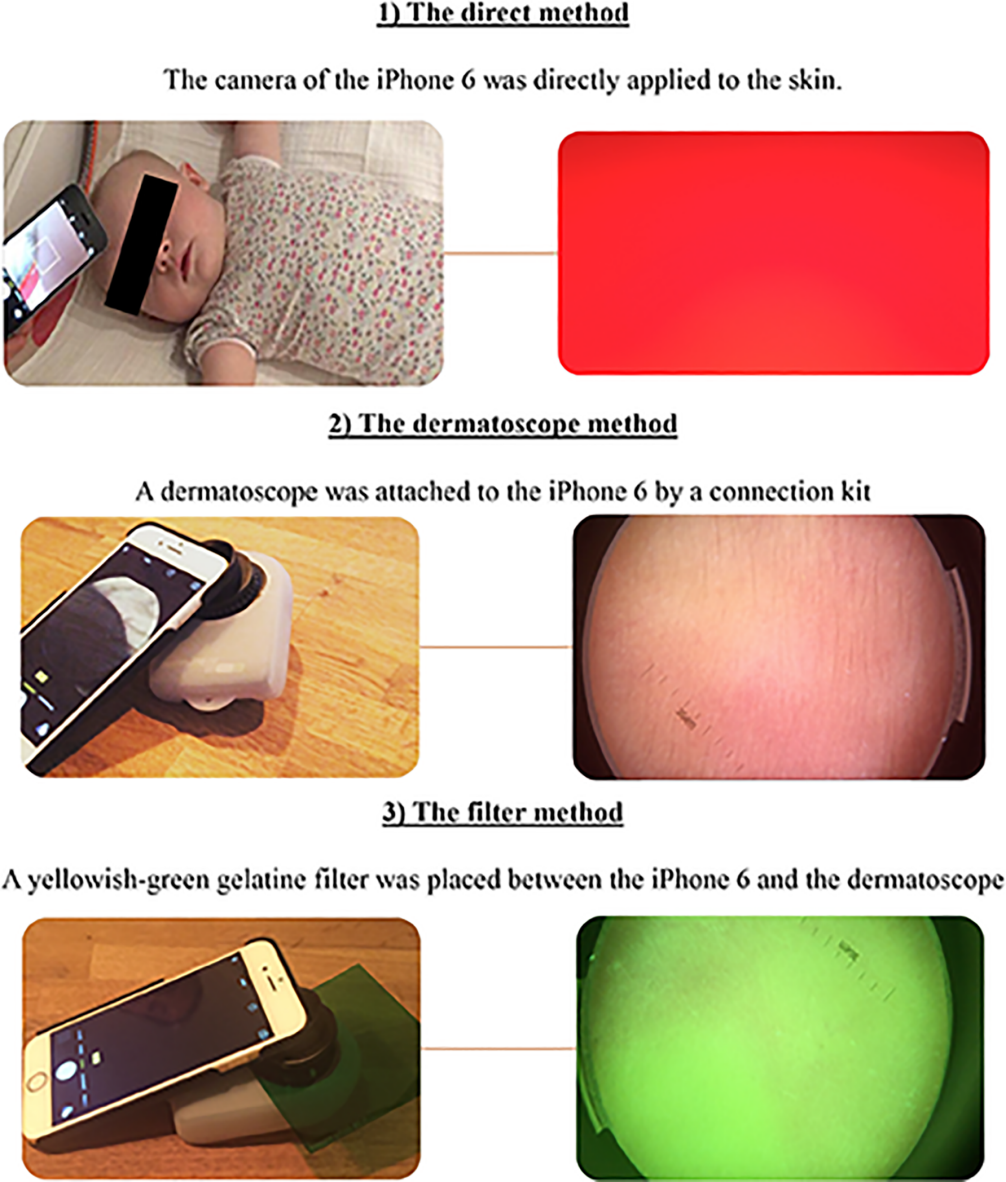
Methods of evaluation. Descriptions of the three methods used in this study. The left picture illustrates the method used to acquire the image, and the right is the resulting image that was used for the data analysis.

This study was approved by the local Ethics Committee of Region North Jutland (Number-201600329) and by the Danish Data Protection Agency. Oral and written consent was obtained from the newborns’ parents before data collection was initiated. Only one pair of parents refused to participate. Written consent to publish the picture of a baby in Fig 1 was achieved from the parents.

### Bilirubin measurement

Blood samples were collected by heel puncture, and TsB levels were analysed in the plasma within two hours with the diazo reaction method on a Cobas 8000 analyser (Roche, Basel, Switzerland). TsB levels were used as a reference and obtained by a single investigator simultaneous with image acquisition. The TcB data were collected using a bilirubinometer (Bilicheck, Philips, Amsterdam, The Netherlands).

### Image acquisition

A single smartphone (iPhone 6; Apple, Cupertino, CA, USA) was used as the primary tool for data collection. The iPhone 6 was equipped with an 8-megapixel iSight-camera with 1.5 μ pixels [17]. The smartphone was disinfected with 70% isopropanol before and after each measurement to avoid any contamination.

We evaluated the following three methods to assess neonatal jaundice, as illustrated in Fig 1: 1) A direct method in which the camera of the iPhone 6 was applied directly to the skin, 2) A dermatoscope method in which a dermatoscope (DermaLite Pro II HR, San Juan Capistrano, CA, USA) was attached to the iPhone 6 with a connection kit, and 3) A filter method in which a yellowish-green gelatine filter (Wratten No. 11, Code: 516FWP7575; Knight Optical, Roebuck, UK) was placed between the iPhone 6 and the dermatoscope. This filter had a wavelength cut-on, peak, and cut-off of 483, 516 and 589 nm, respectively. Theoretically, the filter should reduce the reflectance of haemoglobin, thus enabling the wavelengths produced by bilirubin to be detected [18]. All images were obtained by applying moderate pressure to the glabella of the newborn in order to remove interference produced by the colour of haemoglobin from the superficial skin. While measurements were taken, attention was paid to maintaining constant pressure on the skin. The iPhone was placed in a downward-facing direction. The images were acquired with as little variability as possible while the babies were in a calm state. All the images were taken by one skilled technician in order to reduce variation to a minimum.

### Image analysis

After data collection, a step-by-step review was performed by a single investigator to exclude any inapplicable data, to select the best images and to mark the most uniform area in each chosen image. The average red, green and blue (RGB) channel intensity was calculated for each image in MATLAB R2017a (9.2.0.556344) (MathWorks Inc., Natick, MA, USA). We defined RGB as a colour intensity of 255–0 (R, G, and B) in a digital system with 8 bits per channel.

### Statistics

Calculations were completed using MATLAB, SPSS version 19.0 (IBM, Armonk, NY, USA) or Excel 2017 (Microsoft, Redmond, WA, USA). Pearson’s correlation coefficient (r) was used to evaluate the relationship between each channel intensity and TsB levels. A p value of < 0.05 was considered statistically significant. Outliers were defined as single mean intensity values that deviated more than three standard deviations from the population mean and were extracted from the data set. However, this produced no substantial improvement. Furthermore, the average intensity of 4–16 pixels of each image was recalculated, and the mean, median and central distribution (25 to 75 percentile) of each were subjected to a renewed correlation analyses. Additionally, a stepwise forward multiple linear regression model was performed using RGB intensity and demographic and clinical data as co-variables. However, no improvement over the raw images was achieved.

## Results

The direct and filter method (methods one and three–see Fig 1) produced colour intensity results that were not significantly correlated with bilirubin levels. Therefore, only the dermatoscope method was further evaluated. All data were obtained as both still pictures and videos, but only still pictures are presented because the results obtained from the videos were too variable. Furthermore, the data were compared to both TsB levels and TcB results, but no substantial differences between the two modalities were found. Therefore, the results presented here are compared with only TsB values.

After the data acquired with the dermatoscope method were reviewed, data for 51 out of the 64 neonates were available for the final analysis. Exclusions were usually caused by inadmissible image quality (e.g., the images were not obtained properly or contained too many irregularities, such as hair and skin differences), and in three newborns, TsB data were not available. Demographic and clinical data are shown in Table 1.

**Table 1:** Demographic and clinical data.

Gender, Females/males, n (%)	26/25 (51/49)
Gestational age (weeks), median (range)	40 (35–41)
Birth weight (g), median (range)	3455 (2440–4900)
Postnatal age (days), median (range)	3 (3–9)
Total plasma bilirubin (μmol/L), median (range)	155 (28–289)
Haemoglobin (mmol/L), median (range) (g/dL), median (range)	12.4 (9.1–16.9) 20.0 (14.7–27.2)

Demographic and clinical data for the 51 included newborns who were analysed using the dermatoscope method.

**Fig 2:**
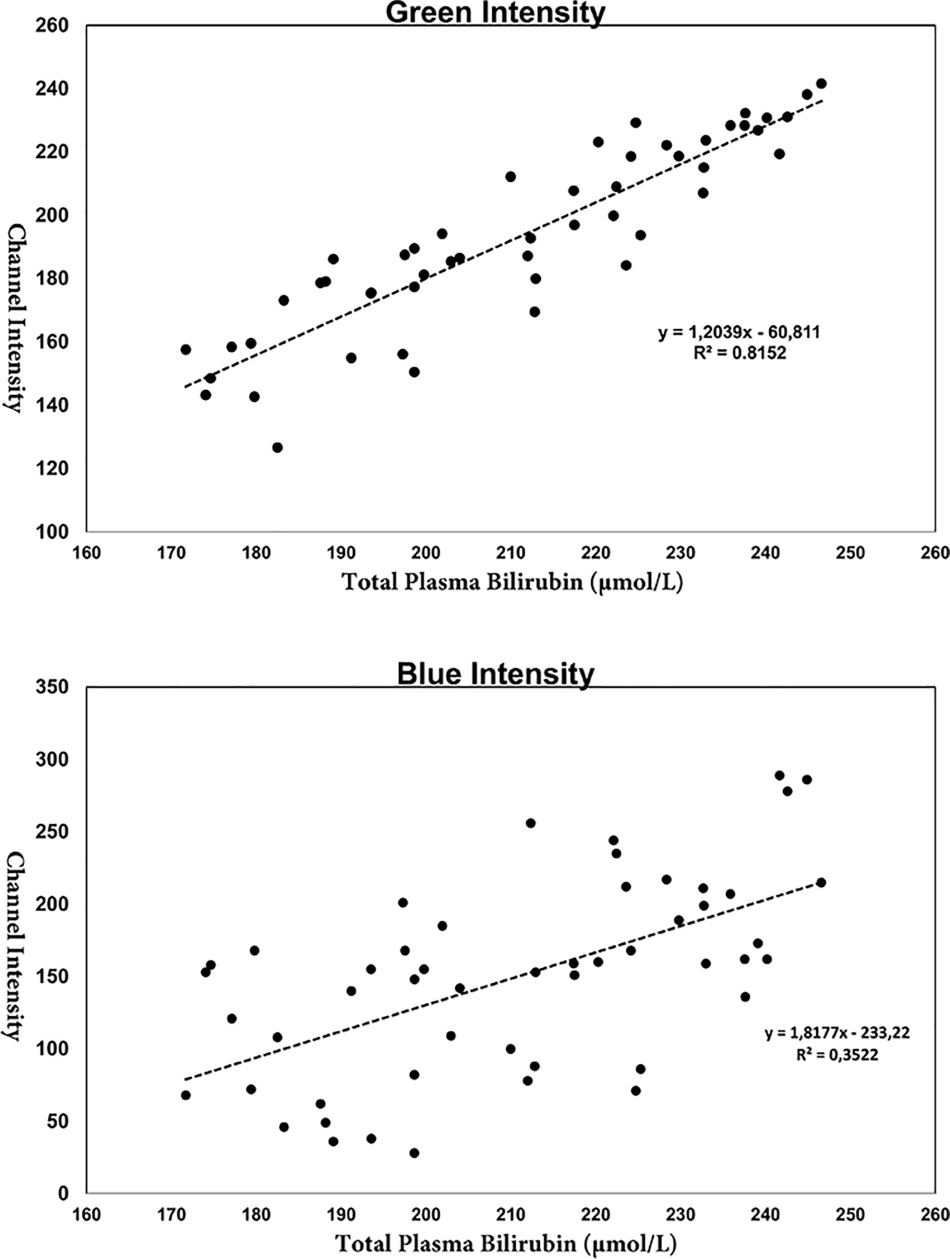
The dermatoscope method; A scatterplot of the green and blue channel intensities and plasma bilirubin. A scatterplot showing the correlation between the green and blue channels using the dermatoscope method and total plasma bilirubin levels.

A Pearson’s correlation coefficient analysis of the red channel resulted in *r* = 0.21 (*p* = 0.14) for the relationship between colour intensity and TsB levels. For the green channel, we found *r* = 0.59 (*p* <0.001), and Fig 2 shows a scatterplot of the relationship between green channel intensity and TsB levels. The result of a Pearson’s correlation analysis of the blue channel was *r* = 0.46 (p <0.001) (Fig 2).

For green channel intensity when using a discrimination limit of 212, 26 newborns had levels above this limit, eleven of whom had TsB > 205 μmol/L, and 25 newborns were below this limit, all of whom had TsB < 205 μmol/L. Therefore, the method had a sensitivity of 100% for identifying newborns with TsB > 205 μmol/L and a specificity of 62.5%. For blue channel intensity, we used a discrimination limit of 190. In this analysis, 10 of 11 newborns had TsB > 205 μmol/L, resulting in a sensitivity of 91% and a specificity of 60%.

## Discussion

In this study, we aimed to determine whether data in images acquired from a smartphone can be used as a screening tool for neonatal hyperbilirubinemia. Several methods were tested, but the dermatoscope method was the only one that produced applicable results. There were significant correlations between the levels of blue and green colour intensities and TsB levels. Moreover, the high sensitivity of this method shows that it has a potential to accurately report whether there is a risk of a bilirubin level being above 205 μmol/L, being a reasonable warning limit, and at the same time 62.5% of newborns with TsB levels below this limit could be excluded. The results of this proof of concept test are therefore supportive of the potentially application of this technique as a screening tool in outpatient monitoring. However, the method itself presents several disadvantages and it is not applicable as a substitute for any current bilirubin-measuring tools at the present stage.

During the past decade, the popularity of ubiquitous smartphones with powerful processors, high-resolution cameras, and communication capabilities has substantially increased [19]. There are currently nearly two billion smartphone users worldwide [20]. These devices therefore represent novel possibilities for functions that facilitate “personalized medicine”, enable tracking of changes over time, and improve patient empowerment and self-management by home-based tests and tools [19–24]. Smartphones may be a unique tool in developing countries because they are highly accessible. Consequently, smartphone-based health-related tools, such as pulse-oximeters and microscopes, have already been developed [1,19,25,26].

This trend has also gained ground in the field of neonatal jaundice. The BiliCam is the first of its kind to use a built-in camera to assess yellowing of the skin according to a colour calibration card, and it has achieved promising results [16,27]. Other tools have been described, but results showing sufficient accuracy have not been published [1,28].

In neonatal jaundice, excess bilirubin in blood vessels diffuses into tissues, leading to yellowing of the skin. The yellowing progresses in the cephalocaudal direction and fades in the opposite direction [29]. Thus, the forehead is a suitable place for transcutaneously detecting bilirubin levels [6,30].The neonatal skin is fairly homogenous compared to adult skin [31]. Several chromophores are present in the skin. These include haemoglobin, bilirubin and melanin, each of which absorbs light at a different wavelength. Bilirubin is a yellow chromophore with a rather broad spectral absorption in the blue region with a maximum around 460 nm. This is utilized in transcutanous reflectance photometry for evaluating bilirubin levels which is in accordance with the fact, that we found the significant correlations between the intensity of the blue and green colour and bilirubin levels. All digital cameras have three types of wavelength-sensitive photodiodes, the combination of which register visible images. The relative intensity from 0–255 of each color sensing diode provide any color. By separating the RGB values specific for the yellow bilirubin, the color of the skin image could be translated into bilirubin levels. The reflectance from haemoglobin was theoretically excluded in the filter method used in this study by the placement of a filter between the dermatoscope and the smartphone camera, but this did not improve the results. The appearance of the skin varies in accordance with several parameters, such as ethnicity, postnatal age, hydration status, epidermal melanin content and cutaneous blood content [6,30–32]. In the statistical analysis of the dermatoscope method, several of these parameters were accounted for in a multiple regression analysis, but the results were not improved.

This study has several limitations. We included a relatively small sample size that consisted of only Caucasian newborns, and only 11 of the newborns had TsB above 205 μmol/L. We did not investigate whether newborns with a high TsB level had a higher intensity of, for example, green light in later pictures if TsB continued to increase. This analysis could have improved the usefulness of data indicating increasing TsB levels. We used only an iPhone 6 as an evaluation tool, and there are probably significant differences between individual devices, models and platforms [15]. We chose the most recently available Apple smartphone because it is the most widely used device in Denmark. However, this is not the case in the rest of the world, where Android smartphones prevail [33]. In addition, smartphone producers make changes to their devices over time that alter camera performance and imaging software. Hence, studies that include smartphone technology would benefit from testing all methods on different devices, models, and platforms.

The tools applied when using the dermatoscope method also present disadvantages and a dermatoscope is not universally accessible. The large surface of the dermatoscope makes it difficult to maintain constant pressure on the glabella. To compensate for this shortcoming, the area that was analysed was manually selected during image analysis. Additionally, the results were impacted to an unknown extent by surrounding light during image acquisition. However, because the principal function of a dermatoscope is presumably to provide a fixed distance from the camera and a consistent light source, this limitation could be improved by applying a more appropriate smartphone attachment and using a technology-based systematic analysis of the image. An alternative device for this could probably be made as a simple and cheap attachment using the flashlight of the camera.

Future studies should aim to develop a connecting intelligent algorithm. Such an algorithm would facilitate screening for inapplicable data and thorough data analysis, and the algorithm could be continuously improved based on interpersonal results as well as communication between users and healthcare facilities [34]. An intelligent software program would enable the resulting data to be analysed in context with other relevant patient data [10,24,35,36]. If the tool could connect to health care facilities and notify them of any adverse outcomes without requiring interpersonal communication, health care staff could take immediate action and thereby potentially reduce the risk of kernicterus and the costs related to it.

In the present study, we used RGB colour intensity because this is a widely used metric for screen image analysis. Shen et al. [15] addressed this limitation and stated that RGB codes are insensitive and application-specific. Thus, using more detailed colour code methods, and using 16-bit RGB values or another more subtractive colour scheme, such as CMYK, could be preferable.

Methods that use a smartphone to perform spectrophotometry in a manner similar to a transcutaneous bilirubinometer have already been established [6]. These technologies may pave the way for new avenues of research to develop more sophisticated tools for measuring neonatal jaundice. However, to our knowledge, no useful method is yet available to perform these tests [19].

## Conclusions

In this study, we performed a proof of concept test that produced results showing that images obtained by a smartphone attached to a dermatoscope that was applied to the glabella of a neonate may provide data useful for screening for neonatal hyperbilirubinemia. However, the current method did not achieve a level of validity acceptable for clinical application. Thus, further elaborations that refine the technique will be needed to make this a reliable tool for screening.

## Supporting information

S1 File Demographic data and clinical data. Demographic and clinical data for all included 64 newborns.
(XLSX)
